# Impact of chemotherapy alone, and chemotherapy plus ipilimumab, on circulating immune cells in patients with metastatic bladder cancer

**DOI:** 10.1186/2051-1426-3-S2-P257

**Published:** 2015-11-04

**Authors:** Matthew D Galsky, Hahn Noah, Alexander Starodub, Ralph J Hauke, Przemyslaw Twardowski, Mark Fleming, Jingjing Qi, Guru Sonpavde, Manishkumar Patel, Jun Zhu, Uma Chippada-Venkata, Costantine Albany, Li Wang, Miriam Merad, William Oh, Nina Bhardwaj, Sacha Gnjatic, Seunghee Kim-Schulze

**Affiliations:** 1 Icahn School of Medicine at Mount Sinai, New York, NY, USA; 2 Mt. Sinai, New York, NY, USA; 3 IU Health Goshen Center for Cancer Care, Goshen, IN, USA; 4 Nebraska Cancer Center, Omaha, NE, USA; 5 City of Hope, Duarte, CA, USA; 6 Mount Sinai School of Medicine, New York, NY, USA; 7 University of Alabama at Birmingham, Birmingham, AL, USA; 8 Indiana University, Indianapolis, IN, USA; 9 Division of Hematology/Medical Oncology, The Tisch Cancer Institute, Icahn School of Medicine at Mount Sinai, New York, NY, USA

## Background

Metastatic bladder cancer (MBC) is a relatively chemosensitive neoplasm yet response durations are generally short-lived. Recently, immune checkpoint blockade has demonstrated unparalleled activity in heavily pre-treated patients (pts) with MBC. The role of standard chemotherapy on the immune system of patients with MBC, and optimal approaches to combining chemotherapy and immune checkpoint blockade, has not been comprehensively explored.

## Methods

Pts with MBC were enrolled on a Phase II trial of chemotherapy + CTLA4 blockade. Patients received 2 cycles of gemcitabine + cisplatin (GC) followed by 4 cycles of GC + ipilimumab (GCI). Flow cytometry was performed on peripheral blood mononuclear cells at baseline, after GC, and after GCI to determine the impact of treatment on the frequency and phenotype of CD4+ and CD8+ T cells, regulatory T cells (CD4+CD25+CD127-CD45RA-Tregs), and myeloid-derived suppressor cells. Comparisons between time-points were made using Wilcoxon's rank test. Plasma collected from patients was assayed for the expression of 41 cytokines and chemokines by multiplex assay at these same timepoints.

## Results

The trial has completed enrollment (n=36) and flow cytometry data are available for the complete treatment sequence on 27 pts as of 5/2015 (Table). Hierarchical cluster analysis of the cytokine/chemokine panel and cellular immunophenotype demonstrated clustering of post-GC alone specimens and post-GC + ipilimumab specimens. The % of CD4+ and CD8+ T cells was significantly increased after addition of ipilimumab. The level of cytokines involved in proinflammatory and T cell activation such as IL-12, IL-7, IL-15, IFNγ, IFNα and IL-1-α and –ß was higher in the post-GC + ipilimumab than those in post-GC.

**Table 1:** 

Immune Cell Subset	Baseline	Post GC	Post GC + Ipi	Wilcoxon signed-rank text (p value)		
				
				Post-GC versus Baseline	Post-GC + Ipi versus Post GC	Post-GC + Ipi verus Baseline
%CD3CD4	8.3 (5.5-13.10)	9.7 (5.1-16.4)	15.8 (9.4-27.0)	0.13	**0.008**	**0.001**
%CD3CD8	4.4 (2.6-6.7)	4.8 (3.1-7.6)	7.3 (4.2-13.5)	0.9	0.06	**0.01**
% Tregs	6.5 (4.1-7.4)	6.2 (5.2-8.8)	6.2 (4.4-8.0)	0.6	0.4	0.8
% Granulcytic MDSC	0.05 (0.01-.1)	0.04 (0.01-0.1)	0.04 (0.01-0.08)	0.7	0.8	0.4
% Monocytic MDSC	0.01 (0.007-0.03)	0.01 (0.004-0.02)	0.02 (0.007-0.03)	0.3	0.09	0.9

## Conclusions

Gemcitabine plus cisplatin alone did not demonstrate significant favorable or unfavorable effects on the circulating immunocytes profiled. The addition of ipilimumab induced pharmacodynamic changes including an increase in circulating CD4+ and CD8+ T cells and modulation of the peripheral blood cytokine/chemokine milieu generally suggestive of an immunostimulatory effect. The immunomodulatory effects of treatment, interpreted in the context of the clinical outcome data, may help refine an understanding of the mechanistic basis of anticancer effects and inform subsequent rational combinations of chemotherapy plus immune checkpoint blockade.

